# Factors Influencing Surgical Outcomes for Intradural Spinal Tumours: A Single-Centre Retrospective Cohort Study

**DOI:** 10.7759/cureus.21815

**Published:** 2022-02-01

**Authors:** Wai C Soon, Benjamin Fisher, Yasir A Chowdhury, James Hodson, Edward Fashola, Ofuchi Egbuji, Andraay Leung, Marcin Czyz, Navin Furtado, Jasmeet Dhir

**Affiliations:** 1 Neurosurgery, Royal Stoke University Hospital, Stoke-on-Trent, GBR; 2 Department of Neurosurgery, Queen Elizabeth Hospital Birmingham, Birmingham, GBR; 3 Institute of Translational Medicine, Queen Elizabeth Hospital Birmingham, Birmingham, GBR; 4 School of Medicine, University of Birmingham, Birmingham, GBR

**Keywords:** extramedullary, intramedullary, tumour, spinal, intradural

## Abstract

Introduction

Intradural spinal tumours are relatively uncommon tumours of the central nervous system. In this study, we sought to assess our current practice and determine the factors which affect the surgical outcomes of intradural spinal tumour resection.

Methods

All consecutive patients who underwent surgical resection of intradural spinal tumours from December 2011 to November 2018 were retrospectively reviewed. The Modified McCormick Scale (MMS) was used to grade patients’ neurological status both pre-operatively and at the latest follow-up. The associations between changes in MMS and variables such as patient demographics, tumour location, number and experience of consultants involved in the procedure, use of intraoperative neuro-monitoring, bony spinal exposure and dural closure methods were assessed. A multivariable binary logistic regression model was performed to identify independent predictors of improvements in MMS. All analyses were performed using IBM SPSS 22 (IBM Corp. Armonk, NY), with p<0.05 deemed to be indicative of statistical significance throughout.

Results

A total of 145 patients met the inclusion criteria, with a median age of 56.5 years; of whom 119 had extramedullary tumours and 26 had intramedullary tumours. Methods of dural closure were variable, and there was an increasing trend over time towards using the laminoplasty approach for bony exposure. Neither the experience of consultants (p=0.991) nor the number of consultants involved (p=0.084) was found to be significantly associated with the change in MMS, with the strongest predictor being the baseline MMS (p<0.001). Patients who had adjuvant therapy were also significantly more likely to have a poorer neurological outcome (p=0.001).

Conclusion

A good neurological baseline is a significant positive predictor of an improved functional outcome. The number and seniority of consultant surgeons involved in intradural spinal tumour resections did not significantly alter the postoperative outcomes of patients in our single-unit retrospective study.

## Introduction

Intradural spinal tumours are relatively uncommon tumours of the central nervous system, and the management of these lesions can be challenging. The operative approach and surgical techniques involved in managing this heterogeneous group of pathological entities can be highly variable, depending on the nature and location of the tumour, alongside surgeon preference and experience.

A post-operative cerebrospinal fluid (CSF) leak is a well-known and common complication of spinal tumour surgery. It is reported in up to 18% of cases and is associated with increased length of stay in the hospital, prolonged bed rest, infection, thromboembolic events and the need for further surgical intervention [1]. There is significant variability in the methods of dural closure and duration of post-operative bed rest - both with the aim of minimising the incidence of CSF leak.

There is evidence in the literature to support that dual-consultant involvement in spinal deformity correction surgery is associated with decreased blood loss, decreased transfusion rate, reduced opiate requirement and shorter hospital stay [2]. In our unit, two-consultant involvement in the surgical management of complex intradural spinal tumours is not uncommon. The objective of this study was to determine the factors which influence surgical outcomes for intradural spinal tumours. Whether the number of consultants involved or their seniority significantly affects surgical outcomes for intradural spinal tumours has yet to be substantiated.

## Materials and methods

A retrospective review of all consecutive patients who underwent surgical resection for intradural spinal tumours from December 2011 to November 2018 was performed. Patients who underwent surgery for infection or lesional biopsy were excluded. Information regarding patient demographics, location of the tumour, number of consultants or trainees directly involved in the procedure, the experience of consultants, surgical approach, intraoperative neuro-monitoring, method of dural closure, duration of bed rest, post-operative complications and outcomes were extracted from electronic medical and operative records. The experience of consultants was stratified into less than or equal to five, six to 10, 11-15 and more than 15 years. The Modified McCormick scale (MMS), as depicted in Table 1 [3], was used to classify patients’ neurological status on a scale of grade I-V, both pre-operatively, and at the latest follow-up.

**Table 1:** Modified McCormick Scale Source: [3]

Grade	Description of Scale
I	Neurologically intact, with normal ambulation, may have minimal dysaesthesia
II	Functional independence, with mild motor or sensory deficit
III	Moderate deficit. Limitation of function but independent with external aid
IV	Severe motor or sensory deficit, limited function and dependent
V	Paraplegia or quadriplegia

Initially, the changes in MMS from the pre-operative assessment to the end of follow-up were assessed using Wilcoxon’s signed-rank test. Patient demographics, surgical factors and short-term outcomes were then compared between consultant-related factors, namely, the number present and the level of experience of the most senior consultant. Nominal factors were analysed using Fisher’s exact tests, with ordinal or continuous variables analysed using the Mann-Whitney U or Kruskal-Wallis test for comparisons across two or more than two groups, respectively. Associations with the change in MMS between the pre-operative assessment and the most recent follow-up were then assessed, using the Mann-Whitney U or Kruskal-Wallis test for nominal factors in two or more than two groups, respectively, and Spearman’s correlation coefficients for ordinal or continuous factors.

A multivariable binary logistic regression model was then performed to identify independent predictors of improvements in MMS. Patients with a pre-operative MMS of 1 were excluded from this analysis since an improvement was not possible in this group. A backwards stepwise approach, with removal at p>0.10, was then used to select factors for inclusion. Any factors with missing data that were not selected for inclusion in the model were removed from consideration, and the analysis was repeated in order to minimise the exclusion of cases. Factors selected for inclusion in this model were then entered into a new model alongside the consultant-related factors. All analyses were performed using IBM SPSS 22 (IBM Corp. Armonk, NY), with p<0.05 deemed to be indicative of statistical significance throughout.

## Results

In total, 145 patients with a median age of 56.5 years (interquartile range [IQR]: 41.3 - 70), of whom 52% were male, met the inclusion criteria and were retrospectively reviewed. For most surgeries, one consultant (82%) and one trainee (78%) were in attendance. Dural closure was the most commonly performed with sutures (67%), with 2% of the procedures using both sutures and non-penetrating titanium clips (LeMaitre Vascular, Burlington, MA). A single procedure used neither sutures nor clips due to a large defect with inadequate dura for closure, but the dura was instead closed with a collagen matrix, Duragen (Integra, Plainsboro, NJ, USA) and fibrin sealant, Tisseel (Baxter, Vienna, Austria). Further details of the cohort and operative approach are reported in Table 2.

**Table 2:** Patient demographics and treatment factors

Factor	Statistic
Age (Years)	56.5 (41.3 - 70.0)
Sex (% Male)	76 (52%)
Pathology	
Extramedullary	119 (82%)
Intramedullary	26 (18%)
Level	
Lumbosacral	5 (3%)
Lumbar	40 (28%)
Thoracolumbar	13 (9%)
Thoracic	53 (37%)
Cervicothoracic	6 (4%)
Cervical	28 (19%)
Re-Do Surgery	9 (6%)
Laminoplasty	18 (12%)
Number of Consultants	
1	119 (82%)
2	26 (18%)
Experience of Most Senior Consultant	
≤5 Years	64 (44%)
6-10 Years	60 (41%)
11-15 Years	13 (9%)
16-20 Years	8 (6%)
Number of Registrars	
0	12 (8%)
1	113 (78%)
2	20 (14%)
Dural Closure [N=125]	
No Suture or Clips	1 (1%)
Suture	84 (67%)
Clips	38 (30%)
Sutures and Clips	2 (2%)
Post-Operative Drain	74 (51%)
Adjuvant Therapy	16 (11%)
Neuro-Monitoring	104 (72%)

A total of 119 (82%) extramedullary and 26 (18%) intramedullary lesions were included in the study. The most common histologies were schwannomas (N=40), meningiomas (N=34), ependymomas (N=14), myxopapillary ependymomas (N=11), and haemangioblastomas (N=4). The remainder (N=42) consisted of a mixture of arachnoid cysts, neuro-enteric cysts, neurofibromas, epidermoid cysts, metastatic lesions, glioblastoma multiforme, haemangiopericytoma, cavernomas, paragangliomas and primitive neuroectodermal tumours.

Post-operatively, patients required a median of two days (IQR: 1-5) of bed rest and had a median length of stay of eight days (IQR: 6-12 days). A total of N=11 (8%) patients developed infections, namely, urinary tract infections (N=5), wound infections (N=4), meningitis (N=1), and pneumonia (N=1). Eight (6%) patients returned to theatre within 30 days, of whom seven underwent surgical treatment of post-operative infection or CSF leak, and one underwent laparotomy for small bowel obstruction (Table 3).

**Table 3:** Post-operative outcomes CSF: cerebrospinal fluid; MMS: Modified McCormick scale

Factor	Statistic
Bed Rest (Days) [N=140]	2 (1-5)
Length of Stay (Days)	8 (6-12)
Wound Leak	3 (2%)
CSF Leak	4 (3%)
Infection	11 (8%)
Return to Theatre (Within 30 Days)	8 (6%)
Duration of Follow-Up (Months)	16.2 (7.3 - 33.5)
Pre-Operative MMS	
1	21 (14%)
2	64 (44%)
3	43 (30%)
4	12 (8%)
5	5 (3%)
MMS at Last Follow-Up [N=144]	
1	60 (42%)
2	45 (31%)
3	30 (21%)
4	6 (4%)
5	3 (2%)
Change in MMS [N=144]	
Better	58 (40%)
Same	79 (55%)
Worse	7 (5%)

**Figure 1:**
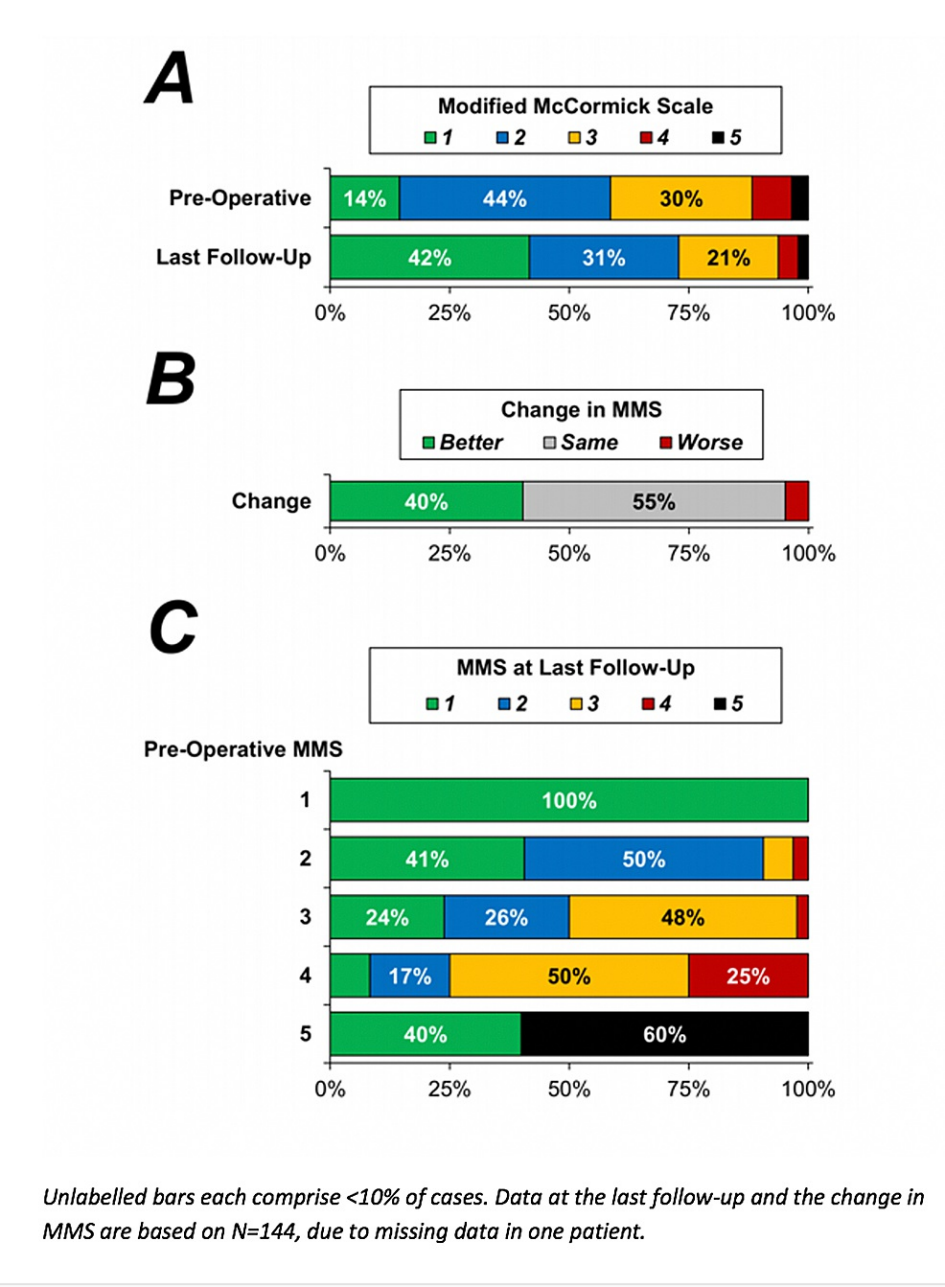
Distribution of MMS scores MMS: Modified McCormick scale

On pre-operative assessment, the majority of patients had an MMS of 2 or 3 (44% and 30%, respectively), with a mean score of 2.4 (Figure 1a). At the most recent follow-up, a median of 16.2 months (IQR: 7.3 - 33.5) after surgery, a significant improvement in MMS was observed (p<0.001), with 40% of patients noted to have improved neurologically by at least one grade of the MMS, and the mean score declined to 1.9 (Figure 1b). Four patients (2.8%) who had resection of an intradural intramedullary tumour died during follow-up, of which three were due to progression of the underlying disease, and the cause of death for one patient who died 32 months after surgery was unknown.

Patient demographics and outcomes were then compared between surgeries with one and two consultants present and with the level of experience of the most senior consultant (Table 4). Patient demographics were generally similar across these groups, with no significant difference in pre-operative MMS detected for either the number (p=0.221) or experience (p=0.532) of consultants. However, less experienced consultants were found to be significantly more likely to perform re-do surgery (13% vs. 5% for ≤5 vs. >10 years’ experience, p=0.010), with patients operated by less experienced consultants having a significantly shorter period of bed rest (median: 2 vs. 5 days, p=0.011).

**Table 4:** Associations between consultants and both patient characteristics and short-term outcomes

	Number of Consultants			Experience of Most Senior Consultant			
	One	Two	p-Value	≤5 Years	6-10 Years	>10 Years	p-Value
Age (Years)	57.5 (41.6-70.5)	50.7 (40.1-66.7)	0.458	55.1 (37.0-70.5)	54.4 (39.8-67.9)	61.0 (49.5-71.4)	0.445
Sex (% Male)	63 (53%)	13 (50%)	0.831	33 (52%)	34 (57%)	9 (43%)	0.541
Pathology (% Extramedullary)	99 (83%)	20 (77%)	0.414	52 (81%)	52 (87%)	15 (71%)	0.268
Level			0.114				0.991
Lumber / Lumbosacral	37 (31%)	8 (31%)		19 (30%)	20 (33%)	6 (29%)	
Thoracic / Thoracolumbar	58 (49%)	8 (31%)		30 (47%)	26 (43%)	10 (48%)	
Cervical / Cervicothoracic	24 (20%)	10 (38%)		15 (23%)	14 (23%)	5 (24%)	
Re-Do surgery	9 (8%)	0 (0%)	0.363	8 (13%)	0 (0%)	1 (5%)	0.010
Laminoplasty	13 (11%)	5 (19%)	0.321	12 (19%)	6 (10%)	0 (0%)	0.056
Number of Trainees			<0.001*				0.023*
0	4 (3%)	8 (31%)		10 (16%)	0 (0%)	2 (10%)	
1	96 (81%)	17 (65%)		47 (73%)	49 (82%)	17 (81%)	
2	19 (16%)	1 (4%)		7 (11%)	11 (18%)	2 (10%)	
Dural Closure***			0.881				1.000
Suture	66 (66%)	18 (72%)		41 (68%)	30 (65%)	13 (68%)	
Clips	31 (31%)	7 (28%)		18 (30%)	14 (30%)	6 (32%)	
Post-Operative Drain	58 (49%)	16 (62%)	0.282	33 (52%)	33 (55%)	8 (38%)	0.403
Adjuvant Therapy	11 (9%)	5 (19%)	0.166	8 (13%)	6 (10%)	2 (10%)	0.935
Neuromonitoring	86 (72%)	18 (69%)	0.811	43 (67%)	45 (75%)	16 (76%)	0.613
Pre-Operative MMS			0.221*				0.532*
1	16 (13%)	5 (19%)		5 (8%)	10 (17%)	6 (29%)	
2	51 (43%)	13 (50%)		30 (47%)	28 (47%)	6 (29%)	
3	37 (31%)	6 (23%)		23 (36%)	14 (23%)	6 (29%)	
4	11 (9%)	1 (4%)		5 (8%)	6 (10%)	1 (5%)	
5	4 (3%)	1 (4%)		1 (2%)	2 (3%)	2 (10%)	
Bed Rest (Days)	2 (1-5)	3 (1-5)	0.639	2 (0-3)	3 (2-5)	5 (0-5)	0.011
Length of Stay (Days)	8 (6-12)	8 (5-14)	0.992	8 (6-14)	8 (6-12)	7 (6-10)	0.392
Wound Leak	3 (3%)	0 (0%)	1.000	2 (3%)	1 (2%)	0 (0%)	1.000
CSF Leak	3 (3%)	1 (4%)	0.551	3 (5%)	1 (2%)	0 (0%)	0.654
Infection	9 (8%)	2 (8%)	1.000	6 (9%)	5 (8%)	0 (0%)	0.441
Return to Theatre (30 Days)	6 (5%)	2 (8%)	0.634	6 (9%)	2 (3%)	0 (0%)	0.262

On univariable analysis, the strongest predictor of the improvement in the MMS was found to be the MMS at the pre-operative assessment (p<0.001, Table 5). However, this was largely since it was not possible for the MMS to improve in those with a pre-operative MMS of 1. Despite this, after excluding these patients, the association between pre-operative MMS and the change in MMS remained significant (p=0.047), with 41%, 50% and 75% of those with a pre-operative MMS of 2, 3 and 4, respectively, seeing an improvement in MMS at the latest follow-up. Further assessment of this association found that, whilst patients with a higher pre-operative MMS were more likely to see some degree of improvement, the MMS at the last follow-up still tended to be higher in these patients than those with a lower pre-operative MMS. For example, whilst those with a pre-operative MMS of 4 were more likely to see an improvement than those with an MMS of 2 (75% vs. 41%), only 8% of those with a pre-operative MMS of 4 had an MMS of 1 at the last follow-up, compared to 41% of those with a pre-operative MMS of 2. This is visualised in Figure 1c.

Of the other factors considered, only adjuvant therapy was found to be significantly associated with the change in MMS, with patients receiving adjuvant therapy being less likely to have an improvement in MMS at the latest follow-up (6% vs. 45%, p=0.001). Neither the number (p=0.084) nor experience (p=0.991) of consultants was found to be significantly associated with the change in MMS in this analysis. Similarly, subgroup analysis within the extramedullary and intramedullary pathologies did not show any significant difference in the change in MMS by either of the consultant-related factors (Table 5).

**Table 5:** Subgroup analysis of the change in MMS by pathology MMS: Modified McCormick scale

Pathology:	Extramedullary (N=118)				Intramedullary (N=26)			
Change in MMS:	Better	Same	Worse	p-Value	Better	Same	Worse	p-Value
Number of Consultants				0.260				0.294
1	44 (44%)	53 (54%)	2 (2%)		7 (35%)	11 (55%)	2 (10%)	
2	6 (32%)	12 (63%)	1 (5%)		1 (17%)	3 (50%)	2 (33%)	
Experience of Most Senior Consultant				0.918				0.959
≤5 Years	21 (41%)	29 (57%)	1 (2%)		4 (33%)	6 (50%)	2 (17%)	
6-10 Years	23 (44%)	27 (52%)	2 (4%)		2 (25%)	5 (63%)	1 (13%)	
>10 Years	6 (40%)	9 (60%)	0 (0%)		2 (33%)	3 (50%)	1 (17%)	

A multivariable analysis was then performed to identify independent predictors of improvements in MMS. Patients with a pre-operative MMS of 1 were excluded from this analysis (N=21), as improvement was not possible in these cases. Of the remainder, 47% (58/123) had an improvement in MMS between the pre-operative assessment and the last follow-up. After considering all factors from Table 6, only adjuvant therapy was found to be a significant independent predictor of the change in MMS, with improvements being significantly less likely in patients that received this (odd ratio: 0.06, 95% CI: 0.01 - 0.48, p=0.008). After accounting for this effect, neither the number of consultants (p=0.565) nor the experience of the most senior consultant (p=0.748) was found to be significantly associated with the likelihood of an improvement in MMS (Table 7).

**Table 6:** Associations with changes in MMS between assessments pre-operatively and at the most recent follow-up MMS: Modified McCormick scale

	Change in Modified McCormick Scale				
	Better	Same	Worse		p-Value
Age (Years)	58 (42-71)	54 (35-70)	60 (48-66)		0.992*
Sex					0.302
Female	30 (44%)	36 (53%)	2 (3%)		
Male	28 (37%)	43 (57%)	5 (7%)		
Pathology					0.098
Extramedullary	50 (42%)	65 (55%)	3 (3%)		
Intramedullary	8 (31%)	14 (54%)	4 (15%)		
Level					0.505
Lumber / Lumbosacral	14 (31%)	30 (67%)	1 (2%)		
Thoracic / Thoracolumbar	27 (42%)	36 (55%)	2 (3%)		
Cervical / Cervicothoracic	17 (50%)	13 (38%)	4 (12%)		
Re-Do surgery					0.753
No	54 (40%)	74 (55%)	7 (5%)		
Yes	4 (44%)	5 (56%)	0 (0%)		
Laminoplasty					0.806
No	50 (40%)	70 (56%)	6 (5%)		
Yes	8 (44%)	9 (50%)	1 (6%)		
Number of Consultants					0.084
1	51 (43%)	64 (54%)	4 (3%)		
2	7 (28%)	15 (60%)	3 (12%)		
Experience of Most Senior Consultant					0.991*
≤5 Years	25 (40%)	35 (56%)	3 (5%)		
6-10 Years	25 (42%)	32 (53%)	3 (5%)		
>10 Years	8 (38%)	12 (57%)	1 (5%)		
Number of Registrars					0.593*
0	5 (42%)	7 (58%)	0 (0%)		
1	46 (41%)	60 (54%)	6 (5%)		
2	7 (35%)	12 (60%)	1 (5%)		
Dural Closure**					0.466
Suture	32 (39%)	45 (54%)	6 (7%)		
Clips	16 (42%)	22 (58%)	0 (0%)		
Post-Operative Drain					0.481
No	29 (41%)	40 (57%)	1 (1%)		
Yes	29 (39%)	39 (53%)	6 (8%)		
Adjuvant Therapy					0.001
No	57 (45%)	66 (52%)	5 (4%)		
Yes	1 (6%)	13 (81%)	2 (13%)		
Neuromonitoring					0.203
No	13 (33%)	24 (60%)	3 (8%)		
Yes	45 (43%)	55 (53%)	4 (4%)		
Pre-Operative MMS***					<0.001*
1	0 (0%)	21 (100%)	0 (0%)		
2	26 (41%)	32 (50%)	6 (9%)		
3	21 (50%)	20 (48%)	1 (2%)		
4	9 (75%)	3 (25%)	0 (0%)		
5	2 (40%)	3 (60%)	0 (0%)		
Length of Follow-Up (Months)	17 (8-39)	17 (7-33)	10 (4-38)		0.167*

**Table 7:** Multivariable analysis of improvement in MMS

	Odds Ratio (95% CI)	p-Value
Number of Consultants (Two)	0.73 (0.25 - 2.15)	0.565
Experience of Most Senior Consultant		0.748
≤5 Years	-	-
6-10 Years	1.26 (0.56 - 2.84)	0.574
>10 Years	1.52 (0.45 - 5.15)	0.506
Adjuvant Therapy (Yes)	0.06 (0.01 - 0.48)	0.008

## Discussion

Spinal surgery is associated with a higher rate of litigation in comparison to other surgical specialities, with the most commonly reported claims related to faulty surgical technique or avoidable surgical error [4]. The ‘Get it Right First Time’ (GIRFT) spinal services report was published in the United Kingdom in 2019 as a national initiative aimed at improving and minimising variation in care provided to patients undergoing spinal surgery [5]. The surgical resection of intradural intramedullary spinal tumours has been highlighted as an area posing a relatively high risk of surgical complications. Furthermore, it has been demonstrated that there remains wide variation in the volume of surgical procedures being performed for intradural spinal tumours across various trusts within the UK. As a result, there has been a drive towards centralising specialist surgical services for these conditions to higher volume centres to improve post-operative outcomes. However, a firm link between higher surgical volumes and improved patient outcomes for these procedures remains to be established [5].

Number of surgeons and surgical outcomes

The introduction of working-hours restrictions for medical practitioners due to the introduction of the European Working Time Directive (EWTD) has led to a knock-on reduction in operative exposure during surgical training. The impact of this is unclear, but it may result in newly appointed consultants being less experienced in managing more complex cases when compared with their predecessors. Babu et al. showed that following the introduction of resident duty-hour restrictions, there was an increased risk of post-operative complications for patients undergoing brain tumour and cerebrovascular procedures [6]. Due to the complexity of certain intradural spinal tumours, it is not uncommon to have joint consultant involvement for complex cases in our unit. There is evidence in the literature to support that by having two consultants operating in complex spinal cases, there is a significant reduction in operative time, peri-operative complications and blood loss [7-8]. In our study, we found that there was no significant correlation between patients’ outcomes and the number of consultants involved. The main drawback for joint consultant cases is the limitation of training opportunities for surgical trainees who would only be involved in more straightforward cases. One should, however, be mindful that there is a great learning opportunity that could be gained from assisting two consultants. A systematic review of the impact of the coronavirus disease 2019 (COVID-19) pandemic on surgical training found that operative experience for trainees has been reduced globally due to a variety of factors such as re-deployment of trainees to other clinical areas of need, cancellation of elective cases and changes to work patterns [9]. In addition, the restriction to the number of surgeons scrubbing in theatre to reduce potential exposure and transmission of coronavirus will also negatively impact surgical training, as trainees may not be able to scrub in cases with joint consultant involvement. Therefore, surgical trainees may find it more challenging to participate and gain competence in the surgical management of complex intradural spinal tumour resection.

Dural closure technique and surgical outcomes

A post-operative CSF leak is a well-recognised complication following spinal surgery, and the reported incidence of CSF leaks requiring secondary intervention varies between 1.7% and 16.7% [10-12]. Patients with CSF leak are at a higher risk of delayed wound healing, infection, neurological deficit secondary to neural compression, and prolonged hospital stay [12]. In our cohort, four patients (3%) returned to theatre for the repair of a post-operative CSF leak. We found that the methods of dural closure used were variable and dural closure using titanium clips was introduced in our unit in 2015. Laut et al. reported that no patients in their series of 50 patients who underwent dural closure using non-penetrating titanium U-clips had post-operative CSF leak [13] and that clips do not cause significant artefacts on post-operative magnetic resonance imaging (MRI) [13]. In another study by Ito et al., the authors found that by using non-penetrating titanium clips, the dura can be closed without creating any holes in the dura, which, in turn, allows the dura to sustain higher leakage pressure when compared with dural closure using sutures [14]. Due to the low incidence of CSF leak in our study (3%), we were unable to compare and determine if there is any significant difference in the incidence of CSF leak between dura closed using clips and sutures. A post-hoc power calculation estimated that, given the incidence and sample size of our study, approximately a seven-fold difference in CSF leak rates, would have been required to detect a difference between closure methods. Assuming a similar overall CSF leak rate (3%), a future study would require approximately 800 patients per closure method to detect a two-fold difference between arms (at 80% power and 5% alpha). However, although we were not able to assess this qualitatively, based on the current literature and our experience, we feel that dural closure using clips is a safe alternative.

Laminoplasty versus laminectomy and surgical outcomes

**Figure 2:**
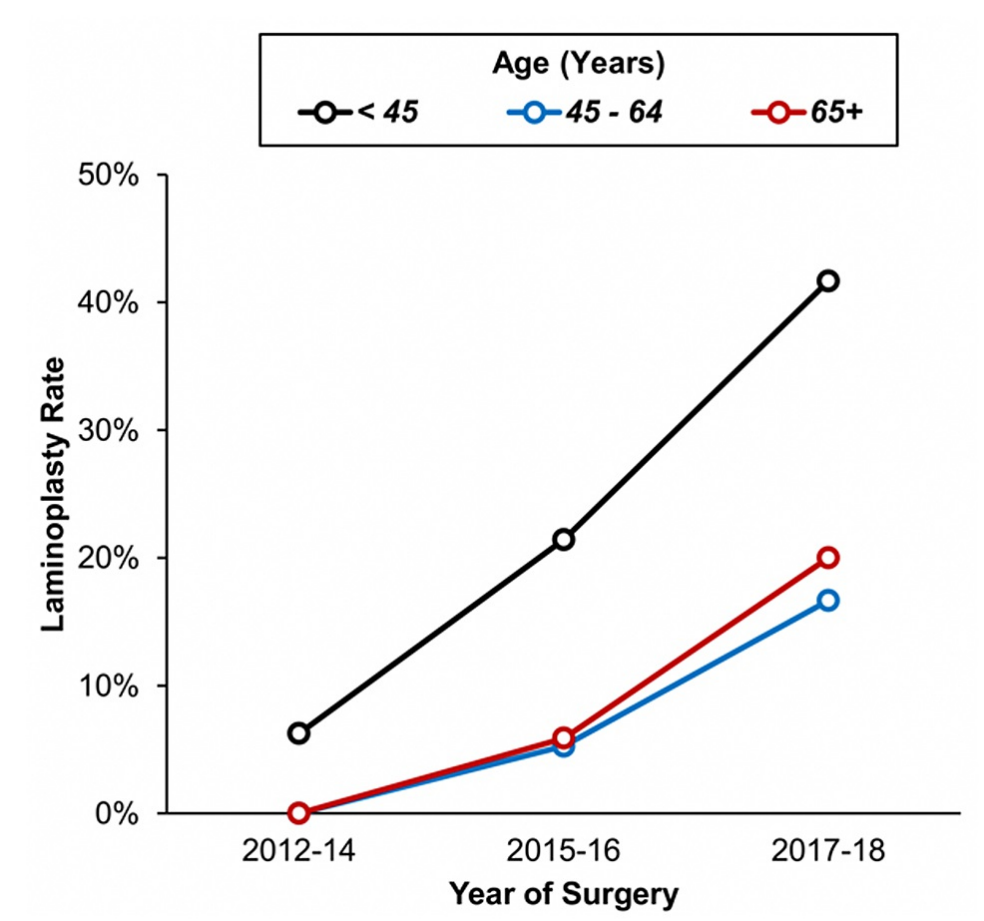
Trend of Laminoplasty Approach

According to the literature, laminoplasty has been proposed to preserve spinal stability and alignment, in addition to protecting the dura from the post-laminectomy membrane, which has been reported to cause neural compression [15-16]. However, its effectiveness in minimising post-operative CSF leak is still unclear. McGirt et al. found that laminoplasty for resection of intradural spinal tumours may be associated with a reduction in incisional CSF leak when compared to laminectomy (3% vs 9%), although the difference was not statistically significant [17]. Interestingly, the authors also found that there is no difference in short-term progressive spinal deformity when comparing laminoplasty versus laminectomy for resection of intradural spinal tumours [17]. In light of the growing body of evidence, there has been an increasing trend towards using the laminoplasty approach for both younger and older patients in our unit (Figure 2), with usage increasing from 6% to 42% in those aged <45 years and 0% to 20% in those aged more than 65 years. This should allow us to assess the long-term outcomes of this approach in the future.

Post-operative bed rest and surgical outcomes

When looking at post-operative bed rest, we found that senior consultants are significantly more likely to keep their patients on bed rest for more than 48 hours. As the incidence of CSF leak in our cohort is low, we are unable to determine whether mandatory post-operative bed rest has a significant impact on the incidence of CSF leak. Low et al. found no significant correlation between the rate of complications and the day of mobilisation following accidental durotomy, and the authors concluded that patients should be mobilised as soon as they are able to [18]. In another retrospective review of 20 patients with incidental durotomy that was managed post-operatively without any mandatory bed rest, the authors found that only one patient had stitch loosening that required revision surgery [19]. Post-operative bed rest has been associated with pulmonary or urinary infections, wound problems like pressure sores or infection, and venous thromboembolism [20]. Radcliff et al. found that patients who are kept on flatbed rest for more than 24 hours are significantly more likely to have medical complications [20]. We feel that early post-operative mobilisation of patients should be encouraged if the operating surgeon is confident with the intraoperative primary closure of the dura with no evidence of CSF egress following Valsalva manoeuvre. This should help minimise post-operative medical complications and reduce the length of stay in the hospital.

Pre-operative baseline and intraoperative neuromonitoring

We found that the strongest predictive factor for a good neurological outcome is the patient’s baseline pre-operative neurological function. This is consistent with the findings of other studies in the literature [21-23]. Therefore, patients with a good baseline neurological function should be offered surgery early, prior to any neurological deterioration. We used intraoperative neuro-monitoring in more than two-thirds of cases and did not find any significant correlation between the usage of intra-operative neuro-monitoring and the neurological outcomes for patients. The use of intraoperative neuro-monitoring has been shown to be an effective tool in anticipating neurological injury during spinal tumour surgery, but it can also limit tumour resection with false readings [24-25]. The D-wave recordings had been found to be the most significant predictor of good motor outcome even when motor-evoked potentials (MEP) are lost intraoperatively [26]. We advocate that D-wave recordings should be used in combination with the MEP recordings, especially during intramedullary spinal tumour resection to ensure maximal safe resection of the tumour.

Study limitations

The findings of our study are limited by its retrospective nature and the heterogeneity of the spinal tumours included for analysis. However, this cohort can well be considered a representative sample of a busy spinal practice within a large UK neurosurgical unit in a teaching hospital. Additional limitations include a potential follow-up bias given the disparate length of time from initial review to outpatient follow-up. Analysis of changes in the MMS outcome could also be potentially affected by the wide range of follow-up times that elapsed between the assessments performed pre-operatively and at the most recent follow-up. As such, if the effect of surgery on symptoms either took some time to manifest or if the benefits of surgery only lasted for a limited period, this effect may have been underestimated in patients with short or long follow-up times, introducing bias into the analysis. To assess the impact of this potential bias, the direction of change in MMS was compared with the duration of follow-up, with no significant association being detected. Hence, it was concluded that the differences in follow-up times were unlikely to have influenced the analysis of the primary outcome.

## Conclusions

Our study demonstrated that the number and seniority of consultant surgeons involved in intradural spinal tumour resections do not significantly alter the post-operative outcomes of patients in our single-unit retrospective study. A good neurological baseline is a significant positive predictor of an improved functional outcome. The MMS grading should form a component of the surgeons’ pre-operative counselling for the treatment of intradural spinal tumours. Other potential factors often cited as important for post-operative outcomes, such as dural closure methods, bony spinal exposure (laminectomy versus laminoplasty) and enforcement of bed rest, were found to have no significant impact upon the post-operative outcome. A larger multi-centre prospective study to further investigate these factors is warranted.
